# Hepatic lesions

**DOI:** 10.1186/1470-7330-15-S1-O28

**Published:** 2015-10-02

**Authors:** Wolfgang Schima, Kartik S Jhaveri

**Affiliations:** 1 Department of Diagnostic and Interventional Radiology, KH Göttlicher Heiland, KH der Barmherzigen Schwestern and Sankt Josef-Krankenhaus, Vinzenzgruppe, Vienna, Austria; 2 Abdominal Imaging, University Health Network, Mt. Sinai and WCH, Toronto, Canada

## 

The liver presents with a variety of lesions for evaluation and appropriate triage with imaging. Ultrasound, MDCT and particularly MRI play a significant role in this objective. In patients without a known malignancy the vast majority of non-cystic lesions are benign (hemangioma, FNH, adenoma, focal fat, etc.), while a few are malignant. However, common benign hepatic lesions may pose a dilemma, if their imaging features are atypical. Although patients with a known malignancy are more likely to have a diagnosis of metastasis for a liver lesion, some studies have shown that small (<1cm) hepatic lesions are more likely to be benign even in patients with a cancer diagnosis [1,2]. While metastases may be a common diagnosis in cancer, it is important to recognise varied patterns of liver metastases after chemotherapy or after surgery. Chemotherapy-related focal or nodular fat deposition can also lead to variety of pseudolesions and one needs to be aware of these appearances and distinguish them from fat-containing hepatic tumors [3]. Uncommon occurrence of hepatic peliosis and sinusoidal obstruction syndrome also needs to be kept in mind in patients with cancer [4].

In patients with chronic liver disease, ultrasound surveillance is the method of choice for the early detection of HCC in cirrhosis [5]. For characterization of focal lesions in cirrhosis, EASL-EORTC and AASLD recommend multi-phasic contrast-enhanced MDCT or MRI. Imaging features typical for HCC is arterial phase hypervascularity and wash-out to hypoattenuation/hypointensity in the venous and/or equilibrium phase, which allows non-invasive diagnosis of HCC [6]. Recently diffusion-weighted imaging (DWI) and liver-specific MR contrast agent have been introduced in the clinical routine for detection and lesion characterization. The combination of DWI and liver-specific contrast agents yields the bests results in the detection liver metastases [7]. For characterization of focal lesions in cirrhosis, administration of liver-specific MR contrast agents may help a make a confident diagnosis [8,9].

In this workshop the work-up of focal liver lesions will be discussed and the varied imaging features of common and less common focal lesions will be presented.
